# Characterization of histopathology and gene-expression profiles of synovitis in early rheumatoid arthritis using targeted biopsy specimens

**DOI:** 10.1186/ar1751

**Published:** 2005-04-25

**Authors:** Takahito Tsubaki, Norimasa Arita, Takuma Kawakami, Takayuki Shiratsuchi, Haruyasu Yamamoto, Nobuo Takubo, Kazuhito Yamada, Sanpei Nakata, Sumiki Yamamoto, Masato Nose

**Affiliations:** 1 Ehime University School of Medicine, Ehime, Japan; 2 Otsuka Pharmaceutical Co Ltd, Tokushima, Japan; 3 Center for Rheumatic Diseases, Matsuyama Red Cross Hospital, Ehime, Japan

## Abstract

The disease category of early rheumatoid arthritis (RA) has been limited with respect to clinical criteria. Pathological manifestations of synovitis in patients whose disease is clinically classified as early RA seem to be heterogeneous, with regular variations. To clarify the relation between the molecular and histopathological features of the synovitis, we analyzed gene-expression profiles in the synovial lining tissues to correlate them with histopathological features. Synovial tissues were obtained from knee joints of 12 patients with early RA by targeted biopsy under arthroscopy. Surgical specimens of long-standing RA (from four patients) were examined as positive controls. Each histopathological parameter characteristic of rheumatoid synovitis in synovial tissues was scored under light microscopy. Total RNAs from synovial lining tissues were obtained from the specimens selected by laser capture microdissection and the mRNAs were amplified by bacteriophage T7 RNA polymerase. Their cDNAs were analyzed in a cDNA microarray with 23,040 cDNAs, and the levels of gene expression in multilayered lining tissues, compared with those of normal-like lining tissues in specimens from the same person, were determined to estimate gene-expression profiles characteristic of the synovial proliferative lesions in each case. Based on cluster analysis of all cases, gene-expression profiles in the lesions in early RA fell into two groups. The groups had different expression levels of genes critical for proliferative inflammation, including those encoding cytokines, adhesion molecules, and extracellular matrices. One group resembled synovitis in long-standing RA and had high scores for some histopathological features – involving accumulations of lymphocytes and plasma cells – but not for other features. Possible differences in the histopathogenesis and prognosis of synovitis between the two groups are discussed in relation to the candidate genes and histopathology.

## Introduction

Synovial lesions in rheumatoid arthritis (RA) show complex histopathological manifestations, involving several diagnostic hallmarks such as multilayered synovial lining tissues associated with a palisading structure of the intimal lining cells and the presence of non-foreign-body-type giant cells, formation of lymphoid follicles, and massive accumulation of plasma cells and macrophages [1]. Mesenchymoid transformation and fibrinoid degeneration are definite histopathological features of RA [2]. These lesions are specific to the synovium in the progression stage of RA and their developmental processes remain unclear.

'Early RA' is a clinical term referring to the early stage of RA used to predict the eventual progression stage of RA. The American College of Rheumatology (ACR) 1987 classification criteria for RA [3] have often been used as a diagnostic tool in patients with recent-onset arthritis. However, these criteria were developed in a population of patients selected according to their disease status to classify rather than to diagnose RA. Thus, the diagnostic usefullness of these criteria in early arthritis is probably not optimal. Likewise, previous histopathological studies have been inconclusive with respect to elucidating histological features typical of early RA [4-6]. Therefore, studies of potential molecular changes in the synovium of patients with early RA may improve our understanding of this disease entity and aid diagnosis in the future.

Biopsy targeting of articular lesions in synovial tissues should be a powerful tool for clarifying the initial events of synovitis in RA. Immunohistochemical analyses of synovitis in RA using targeted biopsy specimens have shown that the histopathological features of synovium in early RA are representative of those in long-standing RA [7,8], suggesting quantitative rather than qualitative differences between various forms of synovitis in RA [9,10]. Laser capture microdissection (LCM) and extraction of total RNA followed by a cDNA microarray are techniques that have been developed mainly in molecular oncology and are used for clarifying molecular markers that have the potential to predict metastasis, sensitivity to drugs, and prognosis [11,12]. The use of these techniques to study the histopathogenesis of the initial step of synovitis in RA and its progression should improve our understanding at the molecular level.

In this study, we focused on the analysis of gene-expression profiles characteristic of proliferative lesions in the synovial lining tissues, which are one of the initial histopathological events of synovitis in early RA. That is, we prepared synovial specimens from early RA by targeted biopsy under arthroscopy, and analyzed gene-expression profiles in the synovial lining tissues selected by LCM in a cDNA microarray by comparing those in multilayered lining tissues with those in normal-like lining tissues in each case. On the basis of a cluster analysis, we propose that the synovial proliferative lesions in early RA can be classified into at least two groups. We discuss the histopathological manifestations characteristic of rheumatoid synovitis in these two groups and also the possible differences in pathogenesis and prognosis of synovitis between them.

## Materials and methods

### Patients and tissue samples

We studied 12 patients with early RA (duration of less than 1 year before the diagnosis), and 4 with long-standing RA (duration of more than 3 years before the diagnosis). Not all patients with early RA could be accurately diagnosed at the time of targeted biopsy, although diagnosis was possible with follow-up assessments. All patients had arthritis of the knee and fulfilled the ACR criteria for RA [3] except E-09 (early RA case no. 9) (see Table 1). Written, informed consent was obtained from each patient before they were entered into the study.

Synovial specimens in early RA were obtained from knee joints by targeted biopsy under arthroscopy, and specimens from long-standing RA were obtained by total knee arthroplasty at the Center for Rheumatic Disease, Matsuyama Red Cross Hospital. The number of specimens obtained from each patient and the macroscopic signs of synovitis with the maximum inflammatory activity at biopsy sites are shown in Table 1. For intraindividual comparison, normal-like synovial specimens that were macroscopically thin and translucent and contained only a few vessels were also obtained from each patient [13].

### Histopathology

One-half of each synovial specimen was used for histopathological analysis. The tissue specimens were fixed with 10% formalin in 0.01 mol/l phosphate buffer, pH 7.2, and embedded in paraffin wax. They were stained with hematoxylin and eosin for examination by light microscopy. Histopathological parameters of synovitis were evaluated in accordance with established criteria [14], with modifications involving the degree of proliferation of synovial cells, typical palisading of synovial cells in the intimal lining layers, non-foreign-body-type giant cells in the lining regions, lymphoid and plasma cell infiltration, neovascularization, mesenchymoid transformation, and fibrinoid necrosis in synovium. Of these features, the degree of proliferation of synovial cells was scored as follows: fewer than three layers (0), three to four layers (1), five to six layers (2), or more than six layers (3). Lymphoid cell infiltration was scored as follows: none to diffuse infiltration (0), lymphoid cell aggregates (1), lymphoid follicles (2), or lymphoid follicles with germinal center formation (3). The other features were evaluated using a quantitative grading system consisting of a 4-point scale: none (0), mild (1), moderate (2), or severe (3). The maximum score with this system was 24. The results of scoring of each histopathological feature are presented as the highest score among all the specimens for the patient. The remaining half of the synovial specimen showing the highest score in the feature 'proliferation of synovial cells' was used as multilayered lining tissue for LCM. Nearly normal synovial tissues from the same patient that had no inflammatory lesions and received a score of 0 for all of the histopathological features were used as 'normal-like lining tissue' for LCM.

### Laser capture microdissection

The tissue samples were placed in embedding medium (Tissue-Tek OCT Compound, Sakura Finetechnical, Tokyo, Japan) and immediately snap frozen in acetone/dry ice in the operating room before transport to the laboratory. All cryoblocks were stored at -80°C until 7-μm-thick cryosections were prepared and mounted on a 1.35-μm-thick polyethylene membrane (PALM, Wolfratshausen, Germany). The sections were immediately fixed for 3 min with acetone and for 1 min with 70% ethanol and then stained rapidly for 1 min with HistoGene™ staining solution (Arctrus, BM Equipment Co Ltd, Tokyo, Japan). They were washed with distilled water and were then dehydrated with 100% ethanol and air-dried with a fan for 3 min.

**Figure 1:**
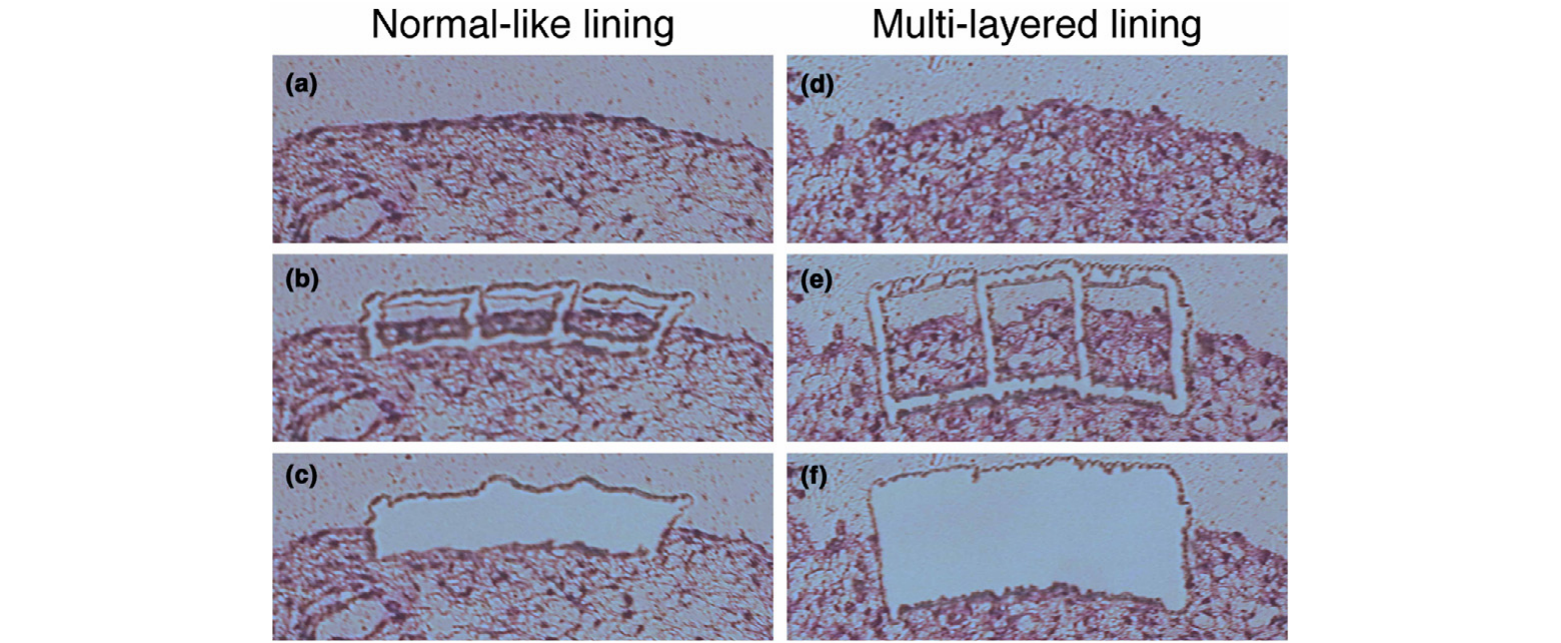
**Laser capture microdissection of synovial lining regions with normal-like lining or multilayered lining. (a,d)**before microdissection; **(b,e) **after tracing around the lining regions together with the intimal lining layer, using a laser microbeam; **(c,f) **catapulted into a microcentrifuge tube by the micromanipulator with a single, precisely aimed laser shot.

LCM was done to collect small regions from a specimen using a Robot-Microbeam (PALM) and an inverted microscope (Carl Zeiss, Oberkochem, Germany) [15]. In brief, the specimen was set on a computer-controlled microscope stage and observed from the upper side with a charged-coupling device (CCD) camera. The image was displayed, and the multilayered lining tissue and the normal-like lining tissue of the same case were selected using the computer mouse (Fig. 1a,d). We traced around the lining and then dissected it to the bottom of the specimen together with the thin membrane, using a laser microbeam through the objective lens (Fig. 1b,e). The selected tissue was then catapulted with a single laser shot into a microcentrifuge cap (0.6 ml), which was held by the micromanipulator (Fig. 1c,f). More than 5,000 cells in each specimen were dissected and pooled for RNA extraction.

### RNA extraction and T7-based RNA amplification

Total RNA was extracted from the samples collected by LCM using an RNeasy spin column purification kit (Qiagen, Hilden, Germany) in accordance with the manufacturer's procedure. To remove possible genomic DNA contamination, RNase-free DNase (Qiagen) was used during the RNA purification steps. Messenger RNA was then amplified by bacteriophage T7 RNA polymerase using a RiboAmp™RNA amplification kit (Arctrus). Two or three rounds of *in vitro *amplification were done with the samples. The amplified RNAs from each multilayered lining tissue and normal-like lining tissue of each case were reverse-transcribed using the SuperScript preamplification system (Life Technologies, Rockville, MD, USA) with random hexamers in the presence of Cy5-dCTP and Cy3-dCTP (Amersham Biosciences Co, Piscataway, NJ, USA), respectively.

### cDNA microarray

A cDNA microarray was fabricated with 23,040 cDNAs selected from the UniGene database of the National Center for Biotechnology . The cDNAs were amplified by RT-PCR using poly(A) + RNAs isolated from various human organs as templates. The PCR products were spotted in duplicate on type VII glass slides (Amersham Biosciences) with a Microarray Spotter Generation III (Amersham Biosciences).

Labeled probes were mixed with Microarray Hybridization Solution Version 2 (Amersham Biosciences) and formamide (Sigma Chemical Co, St Louis, MO, USA) to a final concentration of 50%. After hybridization for 14 to 16 hours at 42°C, the slides were washed for 10 min at 55°C in 2 X saline sodium citrate (SSC) and 1% SDS, for 10 min at 55°C in 0.2 X SSC and 0.1% SDS, and for 1 min at room temperature in 0.1 X SSC. They were then scanned using an Array Scanner Generation III (Amersham Biosciences). The fluorescence intensities of Cy5 and Cy3 for each target spot were evaluated photometrically by the ArrayVision computer program (Amersham Biosciences). Since data derived from low signal intensities are less reliable, a cutoff value for signal intensities of 10,000 was used.

### Cluster analysis

To obtain reproducible clusters for classifying the 16 samples, we selected 1,035 genes for which valid expression data were obtained in all the experiments, and which included an up-regulated (Cy5/Cy3 >2) or down-regulated gene (Cy5/Cy3 <0.5) in at least two of all samples. The analysis was performed using Cluster 3.0 and TreeView software written by M Eisen and updated by Michiel de Hoon, and available on the World Wide Web . Before the clustering algorithm was applied, the fluorescence ratio for each spot was log-transformed (base 2). Then the data were median-centered and normalized for each sample, to remove experimental biases.

### Statistical analysis

Euclidean distance was used to determine the differences between expression levels of individual genes. Statistical analysis on microarray data was performed using the significance analysis of microarrays (SAM) method, available on the World Wide Web . The fold change in expression was calculated for each gene between groups, and significance levels were indicated by the *Q *value. A *Q *value less than 5% was considered significant. A *t*-test was used to confirm the results by SAM. A *P *value less than 0.05 was considered significant. The Mann–Whitney *U *test was used to test for differences in histological scores and disease duration between groups.

## Results

### Histopathological features of synovitis with variations

**Figure 2:**
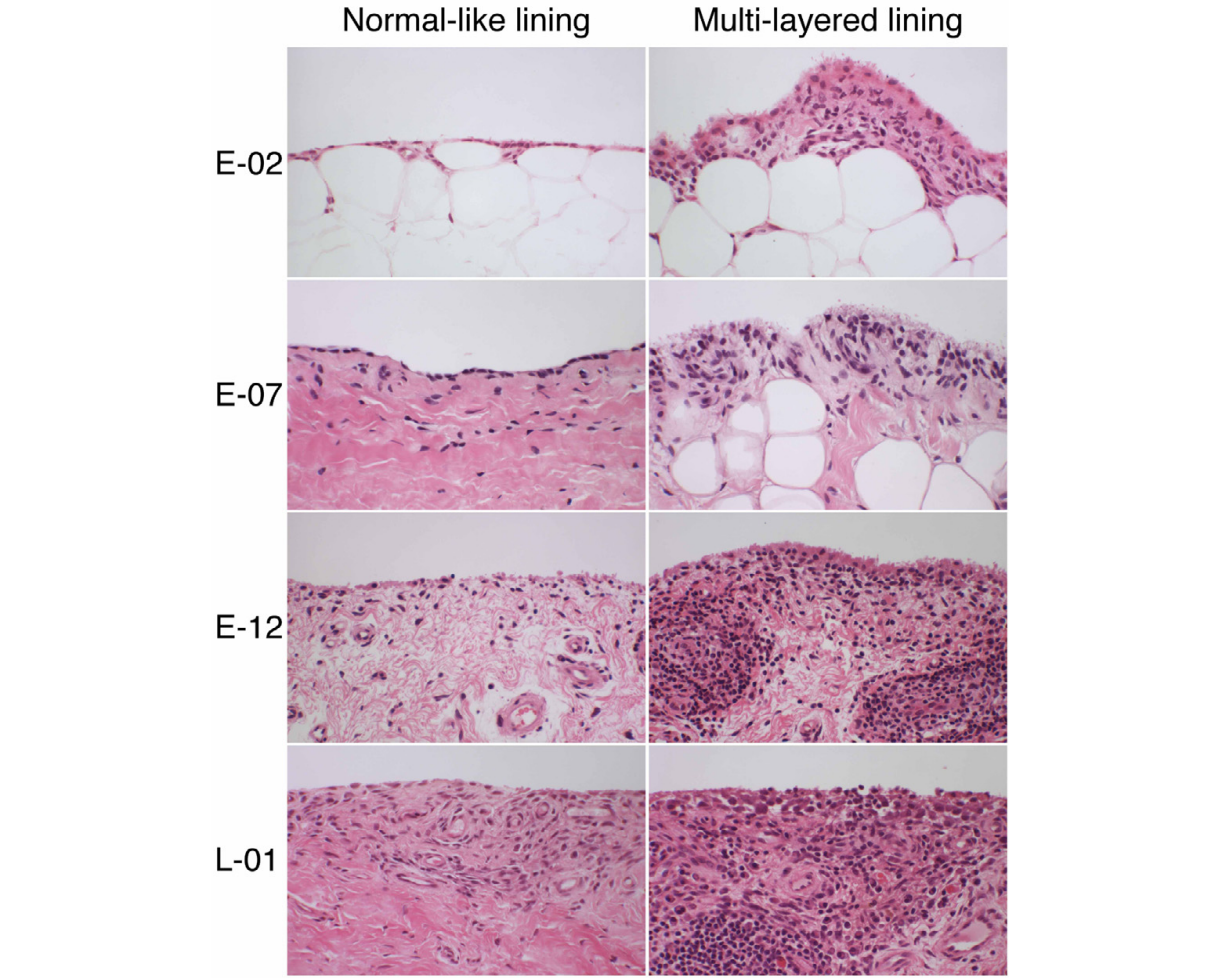
**Histopathological features of synovium in patients with early (E) or long-standing (L) rheumatoid arthritis. (E-02) **The proliferation of synovial lining cells resulted in fewer than four layers. There is diffuse infiltration of macrophages in the sublining regions. **(E-07) **The proliferative lining layer shows a typical palisading structure of the intimal lining layer. **(E-12) **The specimen shows proliferation of synovial lining cells, in places to more than five layers, associated with a typical palisading structure and several non-foreign-body-type giant cells. The lesions manifest underlying proliferation of blood vessels at the arteriole level, associated with many cell infiltrates composed of lymphocytes and plasma cells in the sublining regions. There are foci of lymphocyte aggregates, close to postcapillary venules, resembling lymphoid follicles, but lacking germinal centers. **(L-01) **In contrast to E-12, there are lymphoid follicles with germinal centers.

The histopathology of the early RA specimens showed regular variations. The histological score for each lesion is summarized in Table 2. For example, as shown in Fig. 2, in E-02 the proliferation of synovial lining cells resulted in fewer than four layers (score 1), and a typical palisading structure of the lining cells was not clear (score 1); there was diffuse infiltration of lymphocytes in the sublining regions (score 0). In E-07, the proliferative lining contained fewer than four layers (score 1) but showed a typical palisading structure (score 2).

Some cases of early RA manifested synovitis, in which the histopathological features were similar to those of long-standing RA such as L-01. In E-12, the specimen showed proliferation of synovial lining cells, forming 5 to 6 layers (score 2), associated with a typical palisading structure (score 2), and there were foci of lymphocyte aggregates in the sublining regions, resembling lymphoid follicles but lacking germinal centers (score 1). Many plasma cells were involved in these lesions (score 3) (Fig. 2). Partial fibrinoid necrosis was also present (score 1).

### Gene-expression profiles and clustering

**Figure 3:**
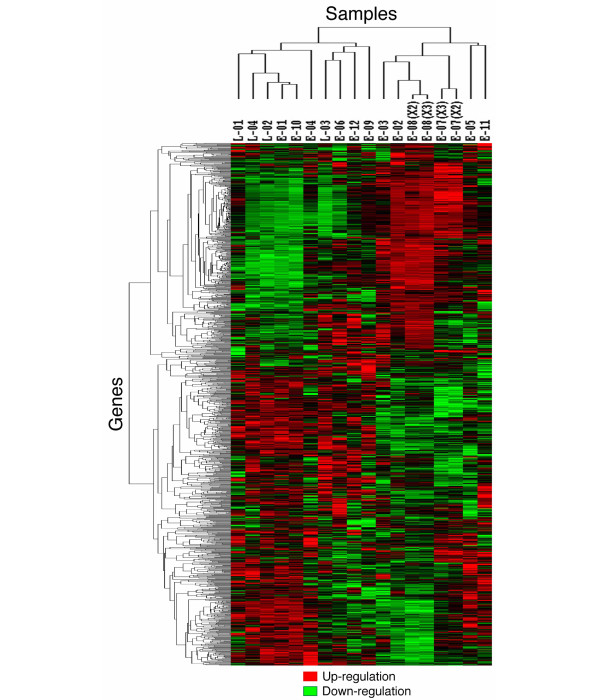
**Dendrogram of two-dimensional hierarchical clustering analysis of 1,035 genes from patients with rheumatoid synovitis. **Red represents relative expression greater than the median expression level among all samples, and green represents relative expression lower than the median expression level. The color intensity represents the magnitude of the deviation from the median. Black indicates unchanged expression. On the horizontal axis, 18 samples from rheumatoid synovitis were clustered into two major groups. On the vertical axis, the 1,035 genes were clustered in different branches according to similarities in their relative expression ratios.

As shown in Fig. 3, 18 samples from 16 cases were clustered into two major groups based on their gene-expression profiles. The dendrogram shown at the top of Fig. 3 represents similarities in expression patterns among individual cases, with shorter branches indicating greater similarities. Two cases (E-07 and E-08), which were examined with two and three rounds of amplification, were clustered most closely, supporting the reliability of our RNA amplification procedures. Of the 16 cases, ten (L-01, L-04, L-02, E-01, E-10, E-04, L-03, E-06, E-12, and E-09) clustered into one group (I) and the other six (E-03, E-02, E-08, E-07, E-05, and E-11) clustered into another group (II). The clustering analysis of only the cases with early RA, not including those with long-standing RA, gave results similar to those shown in Fig. 3. (The result is attached as Additional file 1). Moreover, there was no significant difference in disease duration of the cases with early RA in groups I and II (*P *= 0.34 on the Mann–Whitney test). Each group appeared to have a specific gene-expression profile that should explain the molecular nature of their etiological differences.

### Candidate gene profiles in each group

Using the SAM software, we examined 1,035 genes to find which were expressed significantly differently in groups I and II. We found that the expression of 180 genes was significantly increased and that of 235 was significantly decreased in group II versus group I (*Q *value <5%). From these genes, we selected ones that were of interest on the basis of the data previously reported regarding the mechanisms of rheumatoid synovitis and on the positional candidate genes obtained from our genome data from arthritis models as described in the Discussion. As shown in Table 3A, the genes encoding caspase 9 (*CASP9*), p53 induced gene 11 (*TP53I11*, also called *PIG11*), cathepsin G (*CTSG*), colony-stimulating factor 2 receptor, β (*CSF2RB*), tumor necrosis factor receptor superfamily member 1A (*TNFRSF1A*), and interleukin-10 receptor, β (*IL10RB*) were expressed more abundantly in group II than in group I (*Q *< 5%, *P <*0.05). On the other hand, the genes encoding fibronectin 1 (*FN1*), β2-microglobulin (*B2M*), syndecan 2 (*SDC2*), cathepsin B (*CTSB*), signal transducer and activator of transcription 1 (*STAT1*), integrin, β2 (*ITGB2*), and interferon γ receptor 2 (*IFNGR2*) were expressed more abundantly in group I than in group II (*Q *< 5%, *P <*0.05) (Table 3B).

### Comparative study of histopathological features

There were significant differences in the histological scores of groups I and II (Table 2). The mean total score for group I (13.80) was significantly higher than that for group II (6.67). The mean group I scores for 'typical palisading', 'lymphoid cell infiltration', and 'plasma cell infiltration' were all significantly higher than those for group II. Moreover, in the comparative study of only the cases with early RA, the mean total score and the mean scores for 'lymphoid cell infiltration' and 'plasma cell infiltration' in were significantly higher in group I than in group II. There were no differences between groups I and II in other histopathological features.

## Discussion

There are several reports about gene-expression profiles in rheumatoid synovitis. The analysis by Zanders and colleagues [16] showed an overall increased expression of inflammation-related genes in synovial tissues in RA compared with normal synovium. However, those authors performed the analysis on pooled RA synovial tissues and pooled tissues from healthy controls. Their approach did not consider disease heterogeneity, which may have obscured differences between tissues. Van der Pouw Kraan and colleagues [17] reported that RA synovial tissues could be separated into two patterns of gene expression. The first one had a gene-expression profile consistent with inflammation and active immunity, and the second, which was histopathologically similar to that in osteoarthritis (OA) tissues, exhibited a low level of expression of inflammatory and immune system genes and instead expressed genes related to tissue remodeling. However, their study was performed with whole synovial tissues obtained at synovectomy from long-standing RA and OA patients. Therefore, it may be difficult to use these results to elucidate the developmental process of rheumatoid synovitis.

In this study, we analyzed gene-expression profiles in proliferative lesions of the synovial lining tissues in early RA using targeted biopsy of synovial tissues and LCM, followed by a cDNA microarray. We showed that synovitis in early RA could be divided into at least two different groups based on the gene-expression profiles, although their histopathologies were complex. Group I included the cases with long-standing RA, and some of its synovitis histopathological features were significantly different from those of group II, including lymphoid cell and plasma cell infiltration. Features that seemed to be characteristic of RA, such as synovial cell proliferation in the lining layers, palisading structure of the intimal lining layers, non-foreign-body-type giant cells in the lining regions, neovascularization, and fibrinoid necrosis, were not significantly different in the two groups. On the basis of these findings, we speculate that the two groups may reflect differences in the pathogenesis of synovitis. The different expression profiles of several candidate genes for RA reported previously may support this idea.

### Cytokine networks

Synovial macrophages and fibroblasts in the lining tissue produce factors that activate adjacent cells and enhance synovial inflammation in both paracrine and autocrine fashion [18]. Synovial macrophages activated by tumor necrosis factor α (TNF-α) can increase the production of IL-10. This interleukin has anti-inflammatory effects through its receptor, IL-10R, which is up-regulated on synovial macrophages by TNF-α. IL-10R signaling suppresses the production of IL-1β and TNF-α. The presence of IL-10 may suppress the production of IFNγ by T cells in the synovial tissue [19]. Our study suggests that a negative feedback mechanism by anti-inflammatory cytokines such as IL-10 is predominant in group II, in light of the higher expression of *TNFRSF1A *and *IL10RB *(Table 3A). Thus, IL-10 may play regulatory roles in the progression of synovitis in the early stage of RA.

Synovial macrophages and fibroblasts are strongly activated to express high amounts of IFNγ-inducible genes, despite a low concentration of extracellular IFNγ [20,21]. *STAT1 *is one of the IFNγ-inducible genes. Recently, it was reported that STAT1 protein expression was elevated in rheumatoid synovitis, especially in the lining layer containing highly activated macrophages [17,22]. IFNγ, even in a low concentration, can induce sustained expression of STAT1 through its heterodimeric receptor complex consisting of IFNγ receptors 1 and 2 (IFNGR1 and IFNGR2) [23]. In our study, the signal intensity of *IFNG *itself was very low in all samples (data not shown), while IFNγ-inducible genes such as *STAT1 *and *B2M *were more abundantly expressed in group I (Table 3B). Thus, the effect of IFNγ in rheumatoid synovitis may be evaluated indirectly by the expression profiles of these IFNγ-inducible genes. Considering that infiltrating T cells in the rheumatoid synovium in the early stage of RA are predominantly T helper type 1 cells [8], our findings that the degree of lymphoid cell infiltration was significantly different in the two groups (Table 2) may support this idea.

### Adhesion molecules

There are several histological studies showing the expression of extracellular matrices and integrins in rheumatoid synovitis [24-27]. These adhesion molecules may contribute to a positive feedback mechanism in the cytokine networks [27-29]. In our study, fibronectin 1 was more abundantly expressed in group I than in group II (Table 3B). In the whole genome analysis of rheumatic-disease-susceptibility loci in MRL/lpr mice, *Sdc2 *(encoding syndecan 2) was a candidate gene for progressive arthritis [30]. This was highly expressed in group I in this study. *Itgb2 *was a candidate gene for enthesopathy [31] and coincidentally for sialoadenitis [32], and was also highly expressed in group I.

### Cathepsins

*CTSB*, the gene for cathepsin B, one of the cysteine proteases, was more abundantly expressed in group I than in group II (Table 3B). This protease, which can cleave collagens and proteoglycans, is thought to have a prominent role in destructive arthropathies [33]. It is spontaneously expressed in cultured synovial fibroblasts and can be increased by TNF-α, IL-1, and IFNγ [34,35]. Immunolocalization studies showed cathepsin B to be expressed predominantly in synovial cells attached to the cartilage and bone at sites of rheumatoid joint erosion [33,36]. Taken together, these observations suggest the development of cartilage degeneration and bone resorption in group I, possibly in the progression stage.

On the other hand, *CTSG*, the gene for cathepsin G, one of the serine proteases, was more abundantly expressed in group II than in group I (Table 3A). This protease is normally associated with myeloid cells such as neutrophils and macrophages and can be induced by TNF-α [37]. It has been shown that cathepsin G proteolytically activates caspase 7 [38], an intracellular cysteine proteinase, and, more recently, that it has a role in apoptosis through cleavage of substrates regulating chromatin conformation [39]. This suggests that apoptosis may be impaired in group I.

### p53 tumor suppressor gene

Although RA has many features of autoimmunity, nonimmunologic factors also play a significant role, especially in the progression stage [40-42]. Rheumatoid synovial tissues and synovial fibroblasts exhibit some features of transformation, including autonomous invasion into cartilage, expression of oncogenes, loss of contact inhibition, and insufficient apoptosis [41-44]. p53 protein is induced by many genotoxic stresses, which leads to cell cycle arrest and apoptosis of the injured cells [45]. In our study, *CASP9 *[46] and *PIG11 *[47], which encode proteins involved in apoptosis as downstream targets of p53, were abundantly expressed in group II, but not in group I (Table 3A).

Reactive oxygen and nitrogen species produced at chronic inflammatory sites may damage DNA. If the p53 gene itself gets damaged, apoptosis may be impaired. The p53 mutations are dominant negative and can interfere with endogenous wild-type p53 function [48]. Significantly higher expression of p53 is detected in rheumatoid synovial tissues than in those tissues in patients with OA or reactive arthritis [49]. Of interest, p53 was found in early RA and also in clinically uninvolved joints in RA patients [50]. Yamanishi and colleagues [51] showed that abundant p53 transition mutations, which are characteristic of the DNA damage caused by oxidative stress, were located mainly in the lining tissues, in studies using microdissected rheumatoid synovial tissues. Considering these findings, mutant p53 may be over expressed in the multilayered lining in group I, which fails to induce *CASP9 *and *PIG11*, while wild-type p53 in group II may induce these genes in group II.

The results of the study suggest that a combination of histopathology and gene-expression profiling is a useful tool for diagnostic and prognostic studies of early RA. For example, patients E-01 and E-06 had a few histopathological features specific for RA and showed lower total scores in histopathological features (Table 2), despite the fact that the villous synovial tissues were targeted and examined. However, these patients belonged to group I with respect to their gene-expression profiles. Their disease might advance to the progression stage, the same as the cases of long-standing RA, but different from those in group II. Patient E-05 was a 77-year-old woman who had polyarthralgia associated with marked pitting edema of the dorsum of the hands. The serological tests gave negative results except for mild elevation of erythrocyte sedimentation rate and C-reactive protein. These clinical manifestations could not rule out the possibility of remitting seronegative symmetrical synovitis with pitting edema syndrome (RS3PE) originally described by McCarty and colleagues [52]. This case had a few histopathological features specific for RA except for the proliferation of synovial lining cells associated with a typical palisading structure and it had lower total scores and belonged to group II.

Additional studies will be needed to compare gene-expression profiles of such a case in group II with those of other synovitis diseases such as reactive arthritis and OA, especially with respect to the candidate genes described above. Follow-up studies will be conducted to investigate potential differences in the clinical course of cases in groups I and II.

## Conclusion

In this study, we analyzed gene-expression profiles in the synovial lining tissues *in situ *in early RA using synovial specimens obtained by targeted biopsy, followed by LCM and cDNA microarray analyses. Based on cluster analysis, we found at least two groups in synovitis in early RA, one of which resembled that in long-standing RA. This grouping may reflect differences in the histopathogensis of synovitis in early RA. Different expression profiles of the several candidate genes may provide useful information for future studies of the diagnosis and prognosis of early RA.

## Abbreviations

ACR = American College of Rheumatology; IFN = interferon; IL = interleukin; LCM = laser capture microdissection; OA = osteoarthritis; RA = rheumatoid arthritis; SAM = significance analysis of microarrays; SSC = saline sodium citrate; TNF = tumor necrosis factor.

## Competing interests

The author(s) declare that they have no competing interests.

## Authors' contributions

TT carried out critical examinations in this study, especially synovial targeted biopsy, histopathological analyses, laser capture microdissection, and cluster analysis, and drafted the manuscript as a part of his doctoral thesis, with the assistance of the coauthors. NA prepared histological specimens and carried out laser capture microdissection. TK and TS carried out RNA extraction, the amplification, and a cDNA microarray. HY gave critical suggestions concerning orthopedics. NT, KY, SN, and SY carried out the clinical studies of each case and performed targeted biopsy of synovial tissues with the informed consent of the patients. MN conceived of the study, participated in its design and coordination, and is the corresponding author. All authors read and approved the final manuscript.

## Supplementary Material

Additional File 1 A PDF showing a dendrogram of two-dimensional hierarchical clustering analysis of 1,035 genes among 12 patients with early rheumatoid arthritis (RA), not including the cases with long-standing RA. On the horizontal axis, 12 samples from early RA are clustered into two major groups. The results were similar to those shown in Fig. 3. This may indicate that there was no influence of the cases with long-standing RA in the cluster analysis. Click here for file

Additional File 2 A PDF file showing the results of RT-PCR of multilayered lining tissues. **(A) **Signal intensity of candidate genes in microarray of the four cases of early RA; **(B) **their RT-PCR results. The expression levels of these genes themselves seemed to be well correlated in the two assays. Click here for file

Additional File 3 A PDF file showing dendrograms of two-dimensional hierarchical clustering analysis with two different similarity measures and with two kinds of cutoff value for signal intensities among 18 samples from the 16 cases of rheumatoid synovitis. (Similarity measures: Euclidean distance and Pearson correlation coefficient. Cutoff value for signal intensities: 10,000 and 20,000.) There was no major difference between them regarding the cases belonging to each group. Click here for file

## Figures and Tables

**Table 1:** Characteristics of studied patients with early (E) and long-standing (L) rheumatoid arthritis (RA)

Patient	Age	Sex	Disease duration	ACR criterion nos. fulfilled^a^	Number of samples	Macroscopic signs of synovitis
With early RA						

E-01	51	F	11 months	1, 2, 3, 4	13	Vi, Ve
E-02	50	F	2 months	1, 2, 3, 4, 6	8	Vi, Ve
E-03	34	F	4 months	1, 2, 3, 4, 7	8	Vi, Ve
E-04	34	F	3 months	1, 2, 3, 4, 6, 7	13	Vi, Ve
E-05	77	F	2 months	1, 2, 3, 4, 7	11	Vi
E-06	50	M	4 months	1, 2, 3, 4	11	Vi, Ve
E-07	37	F	7 months	1, 2, 3, 4, 6	6	Ve
E-08	61	F	2 months	1, 2, 3, 4	7	Vi
E-09	75	F	4 months	1, 4, 6	12	Vi, Ve, Gr
E-10	25	F	12 months	1, 2, 3, 4	12	Vi, Ve, Gr
E-11	54	M	12 months	1, 2, 3, 4, 6	11	Ve
E-12	60	F	4 months	1, 2, 3, 4, 6	13	Vi, Ve, Gr

With long-standing RA						

L-01	54	M	9 years	1, 2, 3, 4, 6, 7	6	Vi, Ve, Gr
L-02	77	M	5 years	1, 2, 3, 4, 5, 6, 7	8	Vi, Ve, Gr
L-03	54	F	7 years	1, 2, 3, 4, 6, 7	6	Vi, Ve
L-04	55	F	3 years	1, 2, 3, 4, 6, 7	11	Vi, Ve, Gr

^a^ACR (American College of Rheumatology) criteria: 1, morning stiffness; 2, arthritis of three or more joint areas; 3, arthritis of hand joints; 4, symmetric arthritis; 5, rheumatoid nodules; 6, serum rheumatoid factor; 7, radiographic changes. F, female; Gr, granulation; M, male; Ve, increased number of vessels; Vi, villi.

**Table 2:** Histological scores in patients with early (E) and long-standing (L) rheumatoid arthritis (RA)

	**Group I**										**Group II**					
Histological feature	L-01	L-04	L-02	E-01	E-10	E-04	L-03	E-06	E-12	E-09	E-03	E-02	E-08	E-07	E-05	E-11

Proliferation of synovial cells	3	2	1	1	1	2	2	2	2	2	1	1	2	1	2	1
	1.80 ± 0.63 (1.67 ± 0.52)										1.33 ± 0.52					
Typical palisading	3	3	3	2	2	2	2	1	2	3	1	1	2	2	2	0
	2.30 ± 0.68*(2.00 ± 0.63)										1.33 ± 0.82					
Non-foreign-body giant cells	2	3	3	1	2	1	1	1	2	1	1	3	1	2	0	0
	1.70 ± 0.82 (1.33 ± 0.52)										1.17 ± 0.48					
Lymphoid cell infiltration	3	1	3	0	2	1	2	1	1	2	0	0	0	0	0	0
	1.60 ± 0.97^† ^(1.17 ± 0.75*)										0.00 ± 0.00					
Plasma cell infiltration	3	3	3	0	3	2	3	1	3	3	0	0	1	0	0	0
	2.40 ± 1.08^† ^(2.00 ± 1.27*)										0.17 ± 0.41					
Neovascularization	2	2	2	2	2	2	3	2	2	3	3	3	2	2	1	3
	2.20 ± 0.42 (2.17 ± 0.41)										2.33 ± 0.82					
Mesenchymoid transformation	1	1	2	0	0	0	1	0	0	3	0	0	0	0	0	0
	0.80 ± 1.03 (0.50 ± 1.23)										0.00 ± 0.00					
Fibrinoid necrosis	1	3	2	0	0	0	1	0	1	2	0	0	1	0	1	0
	1.00 ± 1.05 (0.50 ± 0.84)										0.33 ± 0.52					
Total	18	18	19	6	12	10	15	8	13	19	6	8	9	7	6	4
	13.80 ± 4.76^† ^(11.33 ± 4.37*)										6.67 ± 1.75					

The value in the upper row is the histological score of each case. More than 6 samples were taken from each patient for the feature studied. The value in the lower row is the mean ± standard deviation for the group. Values in parentheses (group I) are those for only the patients with early RA. ^†^*P <*0.01, *P <0.05 versus group II on the Mann–Whitney test. ACR, American College of Rheumatology.

**Table 3:** Comparison of the expression of selected genes in two groups of patients with rheumatoid arthritis^a^

Candidate gene	Group I^b^	Group II^b^	*Q *(%)	*P*^*c*^
**A – **Expressed at higher levels in group II than in group I				

*CASP9*	-0.029 ± 0.018	0.020 ± 0.014	0.25	<0.001
*PIG11*	-0.025 ± 0.021	0.024 ± 0.017	0.25	<0.001
*CTSG*	-0.018 ± 0.024	0.031 ± 0.021	0.25	0.001
*CSF2RB*	-0.020 ± 0.024	0.019 ± 0.021	0.61	0.006
*TNFRSF1A*	-0.023 ± 0.029	0.015 ± 0.016	0.77	0.010
*IL10RB*	-0.021 ± 0.027	0.017 ± 0.021	0.90	0.012

**B – **Expressed at higher levels in group I than in group II				

*FN1*	0.023 ± 0.018	-0.030 ± 0.019	0.25	<0.001
*B2M*	0.020 ± 0.023	-0.028 ± 0.022	0.25	0.001
*SDC2*	0.023 ± 0.024	-0.024 ± 0.021	0.25	0.001
*CTSB*	0.006 ± 0.019	-0.036 ± 0.026	0.84	0.002
*STAT1*	0.021 ± 0.031	-0.020 ± 0.014	1.19	0.008
*ITGB2*	0.021 ± 0.025	-0.018 ± 0.023	1.51	0.008
*IFNGR2*	0.019 ± 0.026	-0.016 ± 0.026	3.28	0.022

^a^Statistical analysis on microarray data was performed using the significance analysis of microarrays (SAM) method (see Materials and methods). *Q*, which is the lowest false discovery rate, was considered significant at less than 5%. It is similar to the familiar *P *value, but adapted to the analysis of a large number of genes. ^*b*^Means ± standard deviations for the group, using log-transformed and median-centered microarray data. ^*c*^Values calculated by *t*-test. *P <*0.05 was considered significant.
