# Supplementary figures and images for: Characterization of histopathology and gene-expression profiles of synovitis in early rheumatoid arthritis using targeted biopsy specimens
Source: Arthritis Res Ther. 2005 Apr 25;7(4):R825–36. doi: 10.1186/ar1751 (PMC1175033; doi:10.1186/ar1751)

Genes

Samples

E-03  
E-02  
E-08  
E-05  
E-07  
E-11  
E-04  
E-01  
E-10  
E-06  
E-09  
E-12

Up-regulation  
Down-regulation

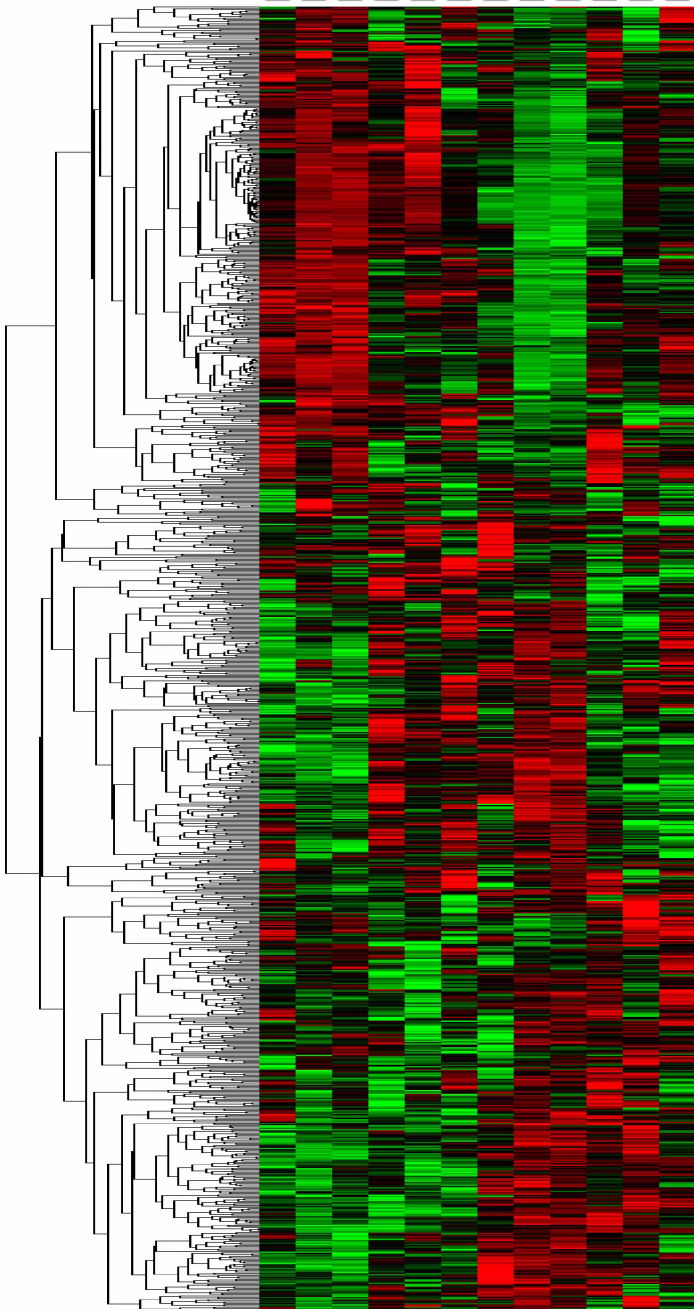

Supplement: Additional File 1 — A PDF showing a dendrogram of two-dimensional hierarchical clustering analysis of 1,035 genes among 12 patients with early rheumatoid arthritis (RA), not including the cases with long-standing RA. On the horizontal axis, 12 samples from early RA are clustered into two major groups. The results were similar to those shown in Fig. 3. This may indicate that there was no influence of the cases with long-standing RA in the cluster analysis. [file ar1751-S1.pdf]

## A. Signal intensity in microarray

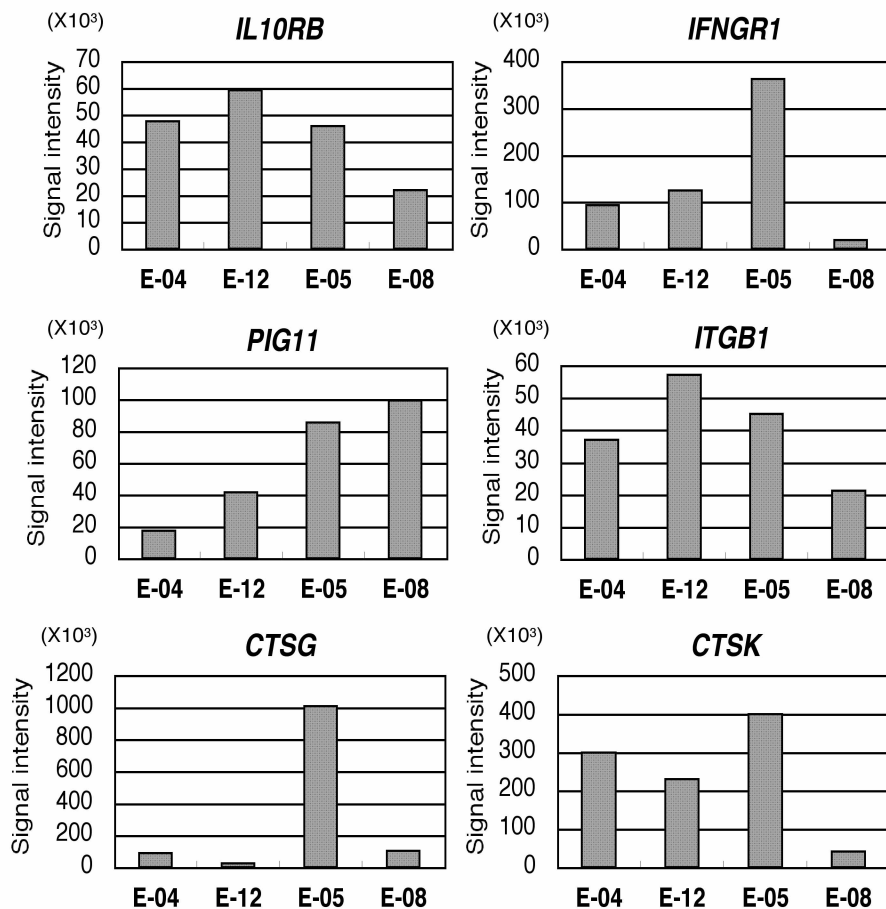

## B. RT-PCR

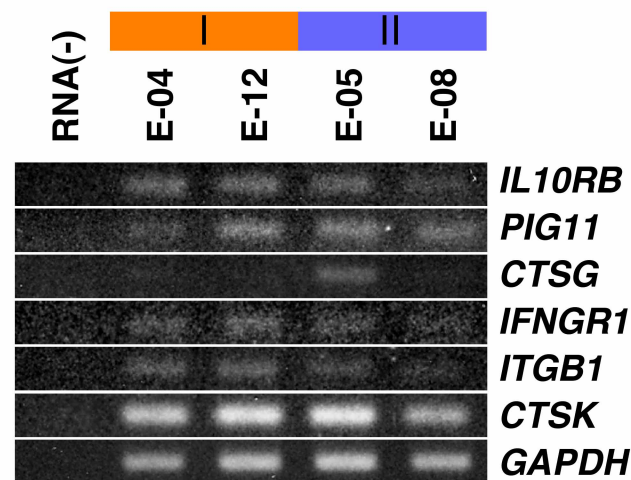

Supplement: Additional File 2 — A PDF file showing the results of RT-PCR of multilayered lining tissues. (A) Signal intensity of candidate genes in microarray of the four cases of early RA; (B) their RT-PCR results. The expression levels of these genes themselves seemed to be well correlated in the two assays. [file ar1751-S2.pdf]
