# Supplementary material for: Characterization of histopathology and gene-expression profiles of synovitis in early rheumatoid arthritis using targeted biopsy specimens
Source: Arthritis Res Ther. 2005 Apr 25;7(4):R825–36. doi: 10.1186/ar1751 (PMC1175033; doi:10.1186/ar1751)
Supplement: Additional File 3 — A PDF file showing dendrograms of two-dimensional hierarchical clustering analysis with two different similarity measures and with two kinds of cutoff value for signal intensities among 18 samples from the 16 cases of rheumatoid synovitis. (Similarity measures: Euclidean distance and Pearson correlation coefficient. Cutoff value for signal intensities: 10,000 and 20,000.) There was no major difference between them regarding the cases belonging to each group. [file ar1751-S3.pdf]

Fig.3

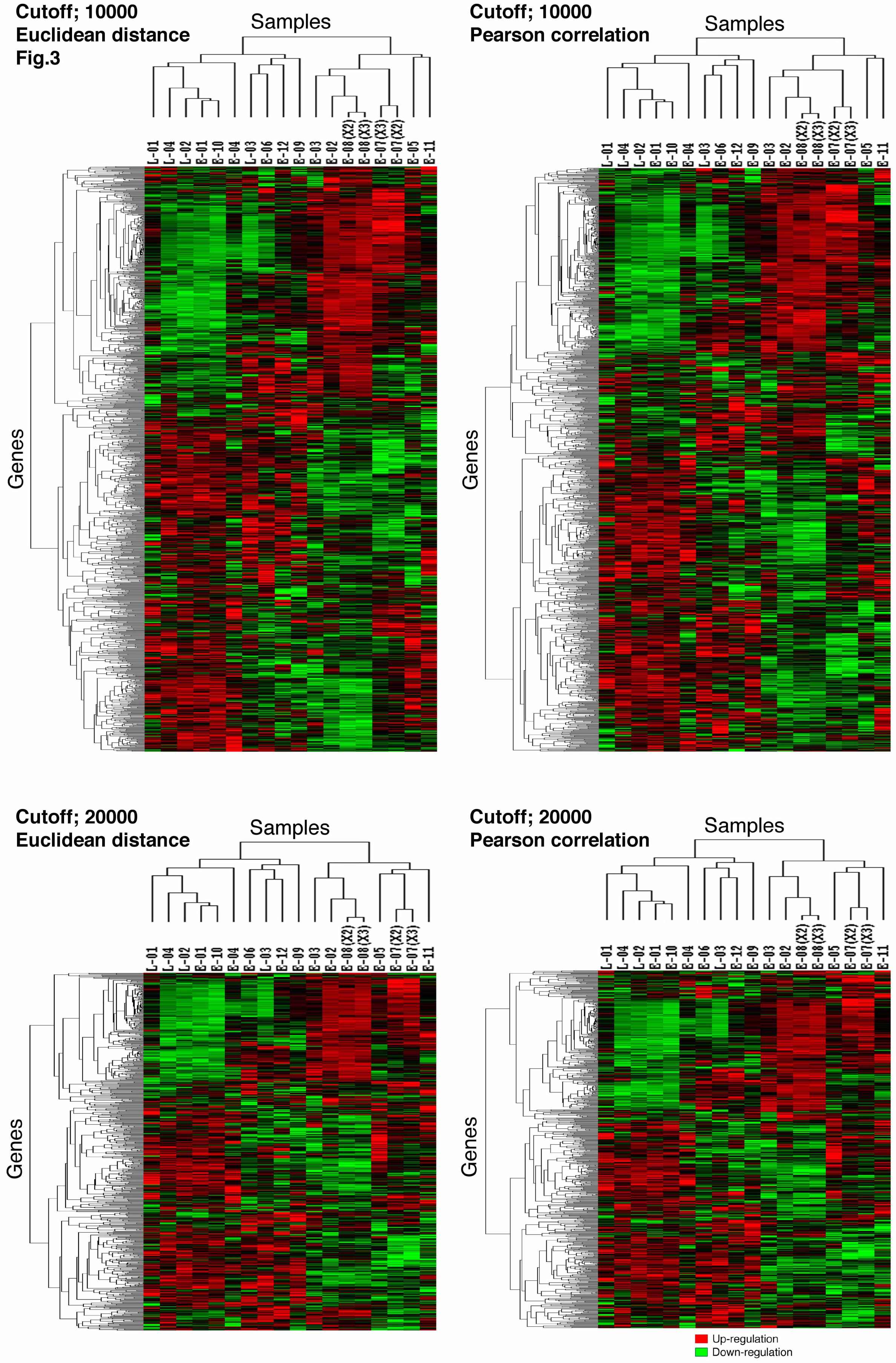
Cutoff; 10000
Euclidean distance
Samples
L-01 L-04 L-02 E-01 E-10 E-04 L-03 E-06 E-12 E-09 E-03 E-02 E-08(X2) E-08(X3) E-07(X3) E-07(X2) E-05 E-11
Genes
Cutoff; 10000
Pearson correlation
Samples
L-01 L-04 L-02 E-01 E-10 E-04 L-03 E-06 E-12 E-09 E-03 E-02 E-08(X2) E-08(X3) E-07(X2) E-07(X3) E-05 E-11
Genes
Cutoff; 20000
Euclidean distance
Samples
L-01 L-04 L-02 E-01 E-10 E-04 E-06 L-03 E-12 E-09 E-03 E-02 E-08(X2) E-08(X3) E-05 E-07(X2) E-07(X3) E-11
Genes
Cutoff; 20000
Pearson correlation
Samples
L-01 L-04 L-02 E-01 E-10 E-04 E-06 L-03 E-12 E-09 E-03 E-02 E-08(X2) E-08(X3) E-05 E-07(X2) E-07(X3) E-11
Genes
Up-regulation
Down-regulation
